# The Role of Gesture in Communication and Cognition: Implications for Understanding and Treating Neurogenic Communication Disorders

**DOI:** 10.3389/fnhum.2020.00323

**Published:** 2020-08-11

**Authors:** Sharice Clough, Melissa C. Duff

**Affiliations:** Communication and Memory Lab, Department of Hearing and Speech Sciences, Vanderbilt University Medical Center, Nashville, TN, United States

**Keywords:** gesture, language, aphasia, traumatic brain injury, right hemisphere damage, Alzheimer’s disease, communication, cognition

## Abstract

When people talk, they gesture. Gesture is a fundamental component of language that contributes meaningful and unique information to a spoken message and reflects the speaker’s underlying knowledge and experiences. Theoretical perspectives of speech and gesture propose that they share a common conceptual origin and have a tightly integrated relationship, overlapping in time, meaning, and function to enrich the communicative context. We review a robust literature from the field of psychology documenting the benefits of gesture for communication for both speakers and listeners, as well as its important cognitive functions for organizing spoken language, and facilitating problem-solving, learning, and memory. Despite this evidence, gesture has been relatively understudied in populations with neurogenic communication disorders. While few studies have examined the rehabilitative potential of gesture in these populations, others have ignored gesture entirely or even discouraged its use. We review the literature characterizing gesture production and its role in intervention for people with aphasia, as well as describe the much sparser literature on gesture in cognitive communication disorders including right hemisphere damage, traumatic brain injury, and Alzheimer’s disease. The neuroanatomical and behavioral profiles of these patient populations provide a unique opportunity to test theories of the relationship of speech and gesture and advance our understanding of their neural correlates. This review highlights several gaps in the field of communication disorders which may serve as a bridge for applying the psychological literature of gesture to the study of language disorders. Such future work would benefit from considering theoretical perspectives of gesture and using more rigorous and quantitative empirical methods in its approaches. We discuss implications for leveraging gesture to explore its untapped potential in understanding and rehabilitating neurogenic communication disorders.

## Introduction

When people talk, they move their hands. Spontaneous hand movements produced in rhythm with speech are called *co-speech gestures* and naturally accompany all spoken language. People from all known cultures and linguistic backgrounds gesture (Feyereisen and de Lannoy, 1991), and gesture is fundamental to communication. Indeed, babies gesture before they produce their first words (Bates, 1976). Congenitally blind speakers who have never seen gesture even gesture to blind listeners (Iverson and Goldin-Meadow, 1997, 1998). Our hands help us talk, think, and remember, sometimes revealing unique knowledge that cannot yet be verbalized (Goldin-Meadow et al., 1993). Everybody gestures, but despite its ubiquity, gesture is often seen as secondary to spoken language, receiving less attention in language research. Gesture is often reduced to a subcategory of non-verbal communication. However, non-verbal does not mean non-language, and theoretical approaches of gesture suggest that speech and gesture arise from the same representational system (McNeill, 1992). In this view, rich conceptual representations contain both imagistic and symbolic information that give rise to gesture and speech, respectively. Both these modalities have communicative functions and originate from the same communicative intention (de Ruiter, 2000).

Gesture serves a variety of functions and overlaps with speech^1^ in both time and meaning. However, gesture differs from speech in notable ways. Gesture conveys information holistically, spatially, and often simultaneously in a single event whereas speech is made up of discrete units that unfold incrementally and sequentially over time to create a cumulative meaning (McNeill, 1992). Throughout this review, we highlight findings that demonstrate that speech and gesture, though integrally related, each have their own unique advantages and affordances; for example, gesture is particularly well-suited for communicating visuo-spatial information which is often omitted from speech entirely. Thus, language research is strengthened by considering both speech and gesture together. The data demonstrate that when taken together, speech and gesture provide a rich communicative context that reflects the cognitive processes that underlie language production, manifesting thought into communication. The study of language has a long history; however, despite proposals that spoken language and gesture either co-evolved (Kendon, 2017) or even that language might have emerged from an earlier gestural communication system (Corballis, 2010, 2012), much of linguistic and psycholinguistic theory has privileged spoken language over multimodal communication. The formal study of gesture in communication is a more recent discipline, gaining traction with the seminal work of McNeill (1992) and since accumulating a robust literature, described below, that details the role of co-speech gesture in a variety of functions in healthy adults for both communication and cognition. However, following the course of linguistics and psycholinguistics, researchers studying language disorders have focused primarily on spoken language, and consequently, we know very little about gesture in these disorders.

Here we provide an interdisciplinary narrative review of the communicative benefits of gesture for both speakers and listeners and its interactions with cognition. Gesture does not only contribute essential information to a message but also actively facilitates the cognitive formation of messages and supports learning and memory. We provide an overview of co-speech gesture theory and describe behavioral evidence of the functions of gesture for communication and cognition across the lifespan. We then discuss the application of this research for studying patient populations with neurogenic communication disorders and identify several gaps for future research. While this review takes great interest in the neurologic representation of gesture in the brain, and specifically the insights that may be revealed by studying gesture in neurogenic communication disorders, studies using electrophysiological and neuroimaging methods are largely excluded and outside of the scope of this review. Rather, we focus on empirical behavioral studies that examine the benefits of gesture on communication, learning, and memory. Thus, this paper aims to highlight the status of gesture in its role for shaping language, cognition, and communication. In doing so, we raise awareness of the extent to which gesture has been understudied in people with neurogenic communication disorders. We review existing literature on the study of gesture in aphasia, for which language impairments are primary, as well as in populations where language impairments are secondary to cognitive deficits, including right hemisphere damage (RHD), traumatic brain injury (TBI), and Alzheimer’s disease (AD). We explore ways in which applying the psychological literature of gesture to neurogenic communication disorders can help us better understand these disorders and leverage gesture for rehabilitation. Such work contributes to our understanding of the neural correlates of gesture to advance theories of co-speech gesture that are psychologically and biologically plausible.

## Theoretical Underpinnings of Speech and Gesture

There has been much theoretical interest in describing the relationship between speech and gesture. These theories either posit that speech and gesture arise from a single conceptual system or that they represent two separate, but tightly integrated systems. One of the first and most influential accounts of gesture production is *The Growth Point Theory* (McNeill, 1992, 2005, 2013; McNeill and Duncan, 2000). To summarize, the growth point is the conceptual starting point of a sentence. It is the initial unit of thought that combines linguistic and imagistic information together to initiate the dynamic cognitive processes that organize thinking for speech and results in co-speech gesture. This theory proposes that speech and gesture originate from a single system where an utterance contains both a linguistic and visuo-spatial structure that cannot be separated. Both speech and gesture, therefore, reflect characteristics of the underlying idea, and one cannot be fully interpreted without considering the other. Speech and gesture are integrated not only at a speaker’s thought conception, but also in perception; listeners integrate information from speech and gesture into a single mental representation. For example, after having watched a storyteller narrate a story, listeners report information from both the storyteller’s speech and gesture in their later retelling (McNeill et al., 1994; Cassell et al., 1999).

Although the majority of speech models do not include gesture, many gesture models are based on Levelt’s (1989) model of speech production where spoken language production occurs in three stages: (1) Representations from long-term memory and knowledge of the communicative context feed into a *conceptualizer* and forms a communicative intention. At this conceptual level, the speaker prepares what they want to communicate and generates a preverbal plan. (2) This information then is passed to a message *formulator* where the lexicon is accessed and grammatical, phonological, and phonetic components are encoded into a linguistic structure. (3) Finally, the message reaches the *articulator* level to produce the planned speech. The message is monitored and refined through feedback mechanisms at various levels. Although speech and gesture take very distinct forms of communication, the pathway that produces them may not be all that different. Both arise from a communicative thought, are shaped and planned, and then motorically executed.

The *Sketch Model* (de Ruiter, 2000) for gesture and speech production is an expansion of Levelt’s classical speech production model and differs from McNeill’s Growth Point Theory in that speech and gesture are described as integrated but separate systems. The *Sketch Model* proposes that gesture and speech follow parallel but separate routes of production, but each originating from one common communicative intention. The *conceptualizer* includes both a preverbal *message* for speech and spatiotemporal *sketch* for gesture that captures aspects of the idea’s size, speed, and location. Thus, speech and gesture are planned together before linguistic formulation occurs. These conceptualizations then diverge, taking one of two routes: the speech formulator or the gesture planner, each of which then develops a motor program to produce overt movement via speech and gesture, respectively. This model would predict that impairments at the conceptual level or communicative intention may affect both speech and gesture production while impairments downstream may have differential effects on speech and gesture production, with either modality able to compensate for the other. This is important because it suggests that gesture may be preserved and therefore, retains its communicative and cognitive functions even in the presence of language or speech disorders. This model was recently revised and renamed the *Asymmetric Redundancy Sketch Model* with modified assumptions that speech is the dominant modality and iconic gestures are mostly redundant with speech content (de Ruiter, 2017; de Beer et al., 2019).

The *Interface Model* (Kita and Özyürek, 2003) is also an extension of Levelt’s (1989) speech production model but proposes that in addition to generating a communicative intention and preverbal plan, the conceptualizer also selects modalities of expression. Speech and gesture then are generated from two separate systems: an *action generator* that activates action schemata for spatial and motor imagery and a *message generator* which formulates a verbal proposition. Critically, these two systems communicate bi-directionally during the conceptualization and formulation of utterances. Thus, gesture is shaped by how information is organized and packaged for speech production as well as the spatial and motoric properties of the referent. Additionally, the *Gesture for Conceptualization Hypothesis* (Kita et al., 2017) proposes that gesture’s base in action schemata has functions beyond organizing utterances for speaking and also mediates cognitive processes, through the activation, manipulation, packaging, and exploring of spatio-motoric information, and thus, has self-oriented functions for both speaking and thinking.

Whether speech and gesture form a single or two tightly integrated systems, it is clear that they are tightly coupled in time (Morrel-Samuels and Krauss, 1992), meaning (McNeill, 1992), and function (Wagner et al., 2014) and are integral parts of the language system. A critical question, then, is how this meaning reaches our fingertips. One possibility arises from the embodied-cognition framework which proposes that all language is grounded in sensorimotor experiences (Zwaan and Madden, 2005; Glenberg and Gallese, 2012). In this view, the gestures we produce reflect sensorimotor experiences and arise from rich memory representations of the world around us. Convergent evidence from behavioral, neuroimaging, and lesion studies support this embodied framework, demonstrating that conceptual representations in the brain are flexible and distributed and dependent on prior perceptual and motor experiences (Kiefer and Pulvermüller, 2012). Motor representations in the brain interact with language; for example, reading action words related to the face, arm, or leg results in activation of the corresponding area of the motor cortex (Hauk et al., 2004), and transcortical magnetic stimulation to motor areas of the arm or leg can increase processing speeds for words like “pick” or “kick,” respectively (Pulvermüller et al., 2005). This link between action and language has important implications for gesture which is motoric in nature and, like speech, stems from rich memory representations and experiences. The *Gesture as Simulated Action* framework (Hostetter and Alibali, 2008, 2010, 2019) proposes that gestures are automatically generated by the mental activations that occur when people think about motor actions and perceptual states and predicts that speakers gesture at higher rates when they activate visuospatial or motor simulations. Indeed, speakers gesture more when retelling a story after watching an animation compared to only having heard it (Hostetter and Skirving, 2011). This model also acknowledges that individual and situational differences in gesture production depend on the speaker’s gesture threshold which can change based on the speaker’s disposition to produce gesture in a particular context. Together, these theories provide compelling support for including gesture in any framework that describes the linguistic system. Next, we consider the broad functions of gesture for communication for both listener and speaker.

## Gesture for Communication

Like the study of spoken language, which can be characterized by its parts (e.g., phonemes, morphemes), the study of gesture has also identified different subtypes of gesture (McNeill, 1992). Broadly, these can be classified as representative or non-representative gestures. Following McNeill’s classification system, representative gestures include *iconic gestures*, which depict the shape, size, action, or position of an object (e.g., the trajectory of a baseball). They also include *metaphoric gestures* which give concrete form to abstract ideas (e.g., a grabbing motion when talking about gaining a run) and *deictic gestures* which are used to refer to the location of an object in space (e.g., pointing to home base while recapping a close play). Non-representative gestures are often called *beat gestures* which are brief, repetitive movements that occur in rhythm with speech but without substantive meaning, serving instead to stress or emphasize certain words (e.g., marking the word “runner” with a wrist flick). Representational gestures are symbolic and can only be interpreted within the context of speech, in contrast to other non-gesture hand movements such as *emblems* which are conventionalized signs (e.g., an umpire crossing and extending his arms to indicate the runner is “safe”) or pantomimes which are imitations of motor actions and can replace speech entirely. Representational gestures are the focus of this paper for the meaningful role they play in spoken language.

### Gesture for the Listener

Perhaps the most obvious communication benefits of gesture are those produced for the listener. While listeners receive much of a message in speech alone, gestures may be particularly communicative in difficult listening situations such as listening in noise (Drijvers and Özyürek, 2017), listening in a second language (Dahl and Ludvigsen, 2014), or listening with hearing loss (Obermeier et al., 2012). However, even in typical listening situations, gestures often communicate unique information that is not present in the speech signal. For example, a speaker might say, “The batter hit the ball,” while gesturing a high arching trajectory, uniquely communicating the ball’s path. In this case, the message cannot be fully understood without integrating speech and gesture. Listeners attend to this unique information in gesture and later report information from both speech and gesture in their retellings (e.g., reporting, “The batter hit a fly ball”). Healthy people integrate information from both speech and gesture into a single memory representation, even when they contain conflicting information (McNeill et al., 1994; Cassell et al., 1999; Smith and Kam, 2012). This is done without explicit awareness or attention to the gestures. In fact, interviewers can mislead eyewitnesses when they gesture during a seemingly open question (e.g., asking, “What was the man wearing?” while producing a hat gesture; Broaders and Goldin-Meadow, 2010).

However, not all gestures are created equal. Although, meta-analyses have found an overall moderate beneficial effect of gesture on listener comprehension (Hostetter, 2011; Dargue et al., 2019), some gestures were more beneficial than others. Gestures improved comprehension most when they were iconic and supplemented speech with unique information. Hostetter (2011) found that child listeners benefited more from gesture than adult listeners; however, a more recent meta-analysis by Dargue et al. (2019) found no significant difference in the benefits of gesture for comprehension between adult and child listeners, indicating that gesture robustly facilitates comprehension across the lifespan. Gesture seems to be particularly important for comprehension when listeners are learning language. Children understand complex syntactic structures (e.g., object-cleft-construction) better when the speaker gestures to help them track referents (Theakston et al., 2014), and children are sensitive to referential gestures, using them to disambiguate pronouns (Smith and Kam, 2015). Adult English-as-second-language learners also demonstrate improved comprehension of lecture material when given access to the teacher’s facial and gesture cues compared to audio-only information (Sueyoshi and Hardison, 2005). Gestures in this study were more helpful for language learners of lower proficiency than high English proficiency speakers, highlighting an important function of gesture in scaffolding language access for both child and adult learners.

Furthermore, speakers design their spoken communication for the listener (Clark and Murphy, 1982), and there is evidence that they intend their gestures to be communicative as well (Goldin-Meadow and Alibali, 2013). Speakers gesture more when their listener can see them (Alibali et al., 2001; Mol et al., 2011), and when explicitly asked to communicate specific information to a listener, speakers frequently provide some of the required information only in gesture (Melinger and Levelt, 2004). Speakers are also sensitive to their listener’s knowledge state and use both more words and gestures when their listener does not share common ground with them (Campisi and Özyürek, 2013; Galati and Brennan, 2013; Hoetjes et al., 2015; Hilliard and Cook, 2016) and produce more iconic gestures to child than adult listeners (Campisi and Özyürek, 2013). When they do share knowledge with a listener, their gestures are less complex and informative (Gerwing and Bavelas, 2004); smaller and less precise (Galati and Brennan, 2013; Hoetjes et al., 2015); and lower in the visual field (Hilliard and Cook, 2016). Thus, speakers design their gestures to illustrate information that is novel or important for the listener, emphasizing the communicative function of gesture.

### Gesture for the Speaker

While it may seem intuitive that gesture has functions for the listener, gesture also has important benefits for the speaker. Although speakers gesture more when their listener can see them (Alibali et al., 2001; Mol et al., 2011), they also produce gestures when the listener cannot. For example, people gesture when talking on the phone (Wei, 2006), and blind speakers even gesture to blind listeners (Iverson and Goldin-Meadow, 1997, 1998). Here we explore the functions of gesture for the speaker.

One view proposes that in addition to communicating information to the listener, gesture plays an active role in speech production. The *Lexical Retrieval Hypothesis* (Krauss, 1998; Krauss et al., 2000) posits that cross-modal priming via gesture increases neural activation and makes words easier to access. Indeed, people gesture more when word retrieval is difficult such as when speaking spontaneously or recalling objects from memory (Chawla and Krauss, 1994; Krauss, 1998; Morsella and Krauss, 2004). The temporal nature of speech and gesture supports this idea as well in that the onset of gesture usually precedes the word with which it is associated (Morrel-Samuels and Krauss, 1992). Furthermore, when gesture is prohibited, people are more dysfluent, exhibiting increased pause time, more filler pauses, and slower speech rate (Graham and Heywood, 1975; Rauscher et al., 1996; Morsella and Krauss, 2004). Krauss et al. (2000) propose that the facilitative effect of gesture happens at the level of the phonological encoder of Levelt’s speech model, where a word’s phonological form is planned for articulation. This proposed mechanism for cross-modal priming is based on “tip-of-the-tongue” studies that have found that word retrieval difficulties are more often phonological rather than semantic in nature (e.g., Jones and Langford, 1987) and that participants experience word retrieval failures when gesture is restricted (Frick-Horbury and Guttentag, 1998; although see Beattie and Coughlan, 1999). Understanding the mechanism of this facilitative effect is critical to applying gesture theory to language interventions for people with neurogenic communication disorders, particularly aphasia for which word finding difficulties are hallmark, a point we will return to later. The Lexical Retrieval Hypothesis proposes that to facilitate word retrieval, gestures should be iconic, representing a generalized semantic feature of the target word (Krauss et al., 2000), for example, gesturing whiskers to retrieve the word “cat.” However, it is unclear how producing gestures related to the conceptual features of a word might directly retrieve the phonological word form. The tip-of-the-tongue phenomenon occurs when a speaker is unable to access stored information in memory but has a “feeling of knowing” (Brown, 1991). During retrieval failure, the speaker often has access to incomplete information about the target word such as the first letter, number of syllables, stress pattern, or part of speech and may be able to identify other words that are phonologically or semantically similar (Brown, 1991). This represents the more abstract lexical representation stage in Levelt’s speech model called the “lemma” which may be a more likely beneficiary of cross-modal priming, where semantic information encoded in gesture may boost specification of the lemma and result in spreading activation for retrieval of phonological form. In contrast to the Lexical Retrieval Hypothesis, other studies have found that speakers gesture more during fluent than disfluent speech and that when speech stops, so does gesture (Mayberry and Jaques, 2000; Graziano and Gullberg, 2018), suggesting that the function of gesture is not compensatory or supportive, but rather it co-produces language together with speech.

Differences between speech and gesture suggest that these modalities may not lend themselves equally well to communicating different kinds of ideas. Given its visual nature, gesture is particularly well-suited to convey spatial information. For example, describing the location of furniture in a room would require more complex descriptions in speech (e.g., “the chair is at a 45-degree angle to the right of couch and facing inward”) than simply demonstrating these relative positions with our hands. Indeed, people gesture more when communicating spatial imagery (Rauscher et al., 1996; Krauss, 1998; Alibali et al., 2001; Alibali, 2005) and describing how to complete motor tasks such as how to wrap a present (Feyereisen and Havard, 1999; Hostetter and Alibali, 2007). It can be difficult to describe such motor tasks at all without moving your hands. In these cases, information is often provided uniquely in the gesture modality and absent from speech. Thus, when communicating complex locations and movements, it is easier to show than tell.

There is also evidence to suggest that gesture facilitates the planning and organization of speech. The *Information Packaging Hypothesis* (Kita, 2000) proposes that gesture plays a role in language production by helping the speaker package visuospatial information into units that are compatible with speech. Indeed, people gesture more when linguistic and processing demands are challenging (Melinger and Kita, 2007; Kita and Davies, 2009). For example, when tasked to describe a complex array of dots, people gestured more when they had to organize the dots themselves in their descriptions compared to people whose dot arrays were “pre-packaged” with connected lines (Hostetter et al., 2007b). Direct evidence for this idea that gesture shapes speech production is demonstrated by manipulating gesture and examining its influence on speech (Kita et al., 2017). Mol and Kita (2012) had participants describe actions involving both manner (e.g., roll) and path (e.g., down) components. In one condition they asked participants to gesture manner and path simultaneously (e.g., making a downward spiraling motion) while in the other condition participants made a separate, sequential gestures for each component (e.g., a turning motion for “roll” and a downward motion for “down”). When participants simultaneously gestured path and manner, they were more likely to verbally produce the information in a single clause (e.g., “It rolled down the hill”) whereas when producing two separate gestures, participants were more likely to produce two clauses (e.g., “It rolled and went down the hill”). Therefore, gestures help to organize spatial information in a way that directly influences how ideas are translated into speech.

In summary, gesture is fundamental to communication, tightly integrated with speech in the formulation and perception of utterances, and often communicates unique information not present in the speech signal, especially about spatial and motoric properties of referents. Thus, speech and gesture each have their own advantages but work together to enrich the language context. Gestures have benefits for both listeners and speakers. Gesture facilitates comprehension, and listeners integrate information from both modalities in their mental representations. Gesture may also facilitate word retrieval and fluency for the speaker and is integrally involved in the process of producing spoken language by helping the speaker package thoughts into units that are compatible with the constraints of speech for a given language system. These same communicative functions of gesture that robustly enrich and facilitate communication in healthy individuals may also extend to people with neurogenic communication disorders as well. Next we review the functions of gesture for cognition.

## Gesture for Cognition

Unlike speech, the spontaneous gestures that speakers produce have no standardized form, but rather, are idiosyncratic. Because they are free to take a variety of forms, they uniquely reveal the speaker’s thoughts in a way speech cannot. The form of our gestures reflects our knowledge and experiences, and increasingly, gesture has been shown to have self-oriented cognitive functions that extend benefits of gesture beyond speaking into cognition more broadly; the *Gesture-for-Conceptualization Hypothesis* (Kita et al., 2017) proposes that gesture facilitates conceptualization by activating, manipulating, packaging, and exploring spatio-motoric information. In other words, gesture helps thinking as well as speaking. Here we explore some of the ways gesture interacts with cognition.

### Gesture Reduces Cognitive Load

Given that speakers gesture more when a task is cognitively or linguistically complex (Melinger and Kita, 2007; Kita and Davies, 2009), it is critical to understand how gesture confers cognitive benefits. One theory is that producing co-speech gesture improves working memory by reducing the cognitive load (Goldin-Meadow et al., 2001). Direct evidence for this hypothesis comes from a dual-task paradigm in which participants are asked to memorize a series of items (such as a string of letters) and then are asked to explain something (e.g., how to solve a math problem) during which gesture is either allowed or prohibited. Afterward, they are tested on recall of the initially learned items. In this task, recall is better for both children and adults when they are allowed to gesture during the explanation phase, suggesting that producing gesture reduces the cognitive load during speaking so that speakers can devote more cognitive resources to rehearsal of the target stimuli (Goldin-Meadow et al., 2001; Wagner et al., 2004; Ping and Goldin-Meadow, 2010). This is especially true when the gestures participants produce are meaningful (Cook et al., 2012). An alternative explanation is that the act of inhibiting gesture production increases cognitive load and reduces performance. Indeed, evidence suggests that inhibiting gestures is more cognitively costly for people with low working memory capacity relative to those with high working memory capacity (Marstaller and Burianová, 2013), and individual differences in working memory abilities predict gesture rate in a story retell task, providing further evidence for a facilitative role of gesture on language production and recall when verbal working memory is taxed (Gillespie et al., 2014). These results highlight the potential benefit of gesture for freeing up cognitive resources, and importantly, suggest potential negative ramifications for restricting gesture use, particularly in special populations that may have reduced working memory or attentional capacities, which is an important consideration in neurogenic communication disorders.

### Spontaneous Gestures Predict Readiness to Learn

Our hands not only reveal what we know but also what we are about to know. Gesture precedes language learning. Children produce their first gesture (typically deictic gestures) between 8 and 12 months prior to their first word at about 12 months (Bates, 1976). Furthermore, the gestures children produce predict which words will enter that child’s vocabulary first (Iverson and Goldin-Meadow, 2005). Before creating multiple-word combinations, babies first combine words with gestures (e.g., pointing at a ball and saying “mine” to communicate “my ball”). Children who produce gesture-word combinations first also produce two-word combinations first (Iverson and Goldin-Meadow, 2005). These early gestures have distal effects on children’s communication as well; gesture use at 14 months predicts vocabulary size at 42 months (Rowe et al., 2008) and 54 months of age (Rowe and Goldin-Meadow, 2009a), and babies who produce more gesture-speech combinations at 18 months of age produce more complex sentences when they are 3-years-old (Rowe and Goldin-Meadow, 2009b).

Gesture continues to predict cognitive development throughout childhood and serves as a cue for when the child is *ready to learn* (Goldin-Meadow et al., 1993). This insight comes from studying young children explaining Piagetian conservation tasks. When explaining these tasks, children gesture frequently. Sometimes, they produce similar explanations in both speech and gesture, but other times, they present an incorrect explanation in speech but convey partial knowledge in gesture (Goldin-Meadow, 2005; Goldin-Meadow and Alibali, 2013). When speech and gesture express different ideas, they are called *gesture-speech mismatches.* Those who produce these mismatches were more likely to benefit from instruction (Church and Goldin-Meadow, 1986). 3rd and 4th graders who produce gesture-speech mismatches when solving mathematical equivalence problems at pretest and learning phases also performed significantly better at post-test than children who did not (Goldin-Meadow and Singer, 2003). In these cases, children convey knowledge with their hands that they may not be able to fully articulate verbally. These gestures reflect transitional knowledge and may reveal that the child is on the cusp of grasping the concept (Perry et al., 1988; Pine et al., 2004). When encouraged to gesture while solving math problems, children produce an even wider range of strategies (Broaders et al., 2007). Similarly, when encouraged to gesture during the Alternative Uses Test, children produced more novel uses for target objects (Kirk and Lewis, 2017); gesture helped them conceptualize different features and uses for objects, some of which could then be verbalized. Thus, gesture use may facilitate creative problem solving and the exploration of ideas (Kita et al., 2017).

Importantly, gesture does not only predict learning in children. Adults produce gesture-speech mismatches during complex spatial and reasoning problems such as when explaining the Tower of Hanoi puzzle (Garber and Goldin-Meadow, 2002), gear movement (Perry and Elder, 1997), algebra (Alibali et al., 1999), and during moral reasoning (Church et al., 1995). Gestures also reveal transitional knowledge during learning of organic chemistry (Ping et al., 2019); when naive adults were asked to solve a set of stereoisomer problems and explain their solutions, all participants produced problem solving strategies in both speech and gesture. However, the researchers found that the participants’ explanations predicted post-test performance only when they demonstrated gesture-speech mismatches in which the relevant strategy was conveyed in gesture. The authors conclude that gesture predicts learning because it reveals implicit knowledge and promotes change. Therefore, gesture depicts transitional knowledge and predicts future learning.

### Gesture Facilitates Memory

Gesture not only depicts a readiness to learn but also makes learning last. Studies of classroom learning have revealed that children learn better (Valenzeno et al., 2003; Singer and Goldin-Meadow, 2005) and show better retention and transfer of new learning (Cook et al., 2013) when their teacher gestures. Furthermore, when teachers gesture a particular strategy during math instruction, children were more likely to produce that gesture themselves during the learning period (Cook and Goldin-Meadow, 2006), possibly mediating or enhancing the effect of teacher gesture on learning. Indeed, although viewing gestures improves learning, producing gestures has an even larger effect on comprehension and memory (Dargue et al., 2019); children learn and remember better when they produce gestures during learning compared to children who spoke only during a lesson (Broaders et al., 2007; Cook et al., 2008; Goldin-Meadow et al., 2009). Therefore, encouraging teachers to gesture improves both children’s access to the information and changes the ways in which they engage and interact with the material themselves.

Gesture facilitates learning and memory in other ways, too. Producing meaningful gestures during foreign language learning (Macedonia and von Kriegstein, 2012; Macedonia, 2014; Sweller et al., 2020) and novel word-learning tasks (Krönke et al., 2013) improves subsequent retrieval. Gesture also seems to facilitate recall of mappings from linguistic representations; when explaining the meaning of metaphors, participants used more detail when allowed to gesture (Argyriou and Kita, 2013; Argyriou et al., 2017), suggesting that gesture helped participants retrieve literal and abstract meanings (Kita et al., 2017). In spatial learning for navigation, participants had better recall for a learned route when they gestured during study phase compared to both mental rehearsal and drawing (So et al., 2014). Furthermore, these same participants demonstrated better learning when they were allowed to gesture during their descriptions at recall. These studies highlight a role of gesture in both linguistic tasks such as word learning as well as non-linguistic tasks such as spatial learning. Thus, gesture leaves lasting traces that affect our representations for language and the world around us.

In sum, in addition to demonstrating benefits for communication, gesture has been shown to serve a variety of cognitive functions, reducing cognitive load to benefit working memory, facilitating the exploration of ideas through transitional knowledge, increasing access to lexical and mental representations, and leading to lasting benefits in learning and memory. Less is known, however, about the neural mechanisms of gesture or how the benefits of gesture for communication and cognition are instantiated in the brain. Likewise, the functions of gesture have been explored to a much more limited degree in individuals with neurologic disorders of language and communication, or neurogenic communication disorders. Yet, the study of gesture in such populations provides a key opportunity to establish, and test, neurobiological models of co-speech gesture. Next we review the existing literature on gesture in these populations.

## Gesture in Neurogenic Communication Disorders

So far, we have reviewed evidence that gesture has robust functions for both communication and cognition. Our hands provide a modality for communicating unique kinds of information, benefiting both listeners and speakers, and they reflect and shape our knowledge and experiences. Despite a rich literature that highlights the benefits of gesture and theorizes a tightly integrated relationship with speech, gesture has received substantially less attention in our efforts to understand and treat neurogenic communication disorders. Here, neurogenic communication disorder is an umbrella term that refers to communication impairments with neurological origin including damage from relatively focal lesions from stroke or diffuse neuropathology from insult or degeneration. The four neurogenic communication disorders reviewed here are aphasia, RHD, TBI, and AD. Of these, aphasia is considered a primary language impairment, often due to focal damage to the canonical language network whereas RHD, TBI, and AD are considered cognitive-communication disorders, where communication deficits are secondary, resulting from primary cognitive deficits (e.g., memory, attention, executive function). Differences among these disordered populations provide key context for testing theories of the relationship between speech and gesture and examining gesture’s role in communication and cognition.

### Aphasia

Aphasia most often occurs after left hemisphere stroke and is defined as a selective and primary language impairment that can result in word-finding deficits (i.e., anomia), impaired grammatical formulation (i.e., agrammatism), and fluency disruptions. Aphasia can affect both expressive and receptive language, and several aphasia subtypes and classification systems exist. However, while there is large variability in aphasia presentation, aphasia has been defined as a disorder of the linguistic system, leaving other forms of cognition intact (although see Martin and Reilly, 2012; Murray, 2012; Fonseca et al., 2017 for examples where cognitive impairments have been identified). Furthermore, people with aphasia (PwA) generally have intact communication, meaning that they know what they want to say, and their intents are pragmatically appropriate. In this case, when people with aphasia are unable to communicate verbally, they continue attempts, often through other modalities, including writing, drawing, and gesture. These forms of communication are encouraged in therapeutic approaches prioritizing functional communication.

Gesture research in aphasia has largely examined gesture in three ways: characterizing gesture use, inhibiting gesture use to rehabilitate spoken language, and encouraging gesture use to facilitate functional communication. As reviewed above, healthy adults produce rich spontaneous gestures that take a variety of forms and communicate unique information that supplements the speech signal. These gestures depict spatio-motoric properties that are not easily expressed in language. Gesture is a ubiquitous and natural part of communication, and it is worth exploring how gesture is affected by language disorders and whether it can support, or hinder, recovery.

#### Characterizing Gesture Production

Early studies have primarily characterized gesture production of PwA to see whether language deficits extended to a similar disruption in the manual modality (see Rose, 2006, for a historical review). These studies confirmed that PwA do indeed gesture (e.g., Herrmann et al., 1988; McNeill, 1992; Goodwin, 2000). However, their gestures seem to differ from those of non-brain-damaged individuals. While people with aphasia produce a lower rate of gestures per minute than healthy comparison participants (Cicone et al., 1979; McNeill, 1992), likely due to also producing fewer words per minute, they produce a higher rate of gestures per word (Feyereisen, 1983; Carlomagno and Cristilli, 2006; Sekine et al., 2013; de Beer et al., 2019) and a larger variety of gesture types than healthy participants (Sekine and Rose, 2013). Gesture production also seems to vary by type of aphasia and on the dimension of fluency; Cicone et al. (1979) found that gesture form parallels verbal output where people with non-fluent aphasia produced fewer but clear and informative gestures, and people with fluent aphasia produced frequent but vague gestures. In contrast, other studies have found that people with non-fluent aphasia gesture at higher rates than those with fluent aphasia (Kong et al., 2017). In a story retell task, people with non-fluent Broca’s aphasia produced almost twice as many gestures per 100 words as people with fluent Wernicke’s aphasia, and they also differed by gesture type; people with Broca’s aphasia were more likely to produce meaningful gestures such as iconic gestures whereas those with Wernicke’s aphasia produced more beat and metaphoric, or abstract, gestures (Sekine et al., 2013). However, while people with Broca’s aphasia seem to produce more iconic gestures per word, those with Wernicke’s aphasia produce more iconic gestures per unit of time (Carlomagno and Cristilli, 2006). Critically, brain lesions resulting in aphasia also frequently produce contralateral hemiparesis or limb apraxia, restricting limb use and thus potentially impacting gesture (see Rose, 2006, for a review of the impact of limb apraxia on gesture production). However, studies comparing people with aphasia with and without hemiparesis have found no difference in the number of gestures per word produced (Kong et al., 2015) or the comprehensibility of the gestures produced (Hogrefe et al., 2012, 2017).

One explanation for the increased gesture use by PwA is that it is used to replace speech, serving a compensatory function when verbal communication fails, which accords with theoretical models of speech and gesture that posit highly integrated prelinguistic origins of speech and gesture (for a discussion, see de Ruiter and de Beer, 2013). Behrmann and Penn (1984) found that the functions of gesture production also differed by fluency; people with non-fluent aphasia primarily used gesture to substitute verbal communication while those with fluent aphasia used it to support verbal communication. In conversational speech, 20% of the gestures made by PwA were considered essential (i.e., conveyed information not present in speech) compared to a minimal number of essential gestures produced by healthy comparison participants (van Nispen et al., 2017). Furthermore, Dipper et al. (2015) examined the narrative retellings of PwA and comparison participants describing key motion events from a cartoon depicting the actions “swing” and “roll.” PwA were more likely than healthy comparisons to produce gesture-speech mismatches with a semantically light verb in speech (e.g., “go” for “swing”) and a semantically richer verb in gesture (e.g., gesturing an arc-shaped trajectory), carrying more weight in the gesture modality. Thus, the use of gestures by PwA has a clear communicative function. In fact, listeners more accurately interpret PwAs’ message when provided both speech and gesture video compared to an audio only signal (De Beer et al., 2017; Rose et al., 2017), suggesting that PwA rely more on gestures to communicate their message relative to healthy adults.

Another explanation for increased gesture use is that, consistent with the *Lexical Retrieval Hypothesis* (Krauss, 1998; Krauss et al., 2000), PwA gesture to resolve anomia. Analyzing the frequency of gesture production, Cocks et al. (2013) found that although PwA produced more iconic gestures than control participants, the frequency of iconic gesture did not differ between the two groups when gestures produced during word retrieval difficulties were removed. In conversational samples, PwA produced significantly more gestures during word retrieval difficulty (69%) compared to fluent speech production (31%), and 93.8% of the gestures PwA produced during word retrieval were meaningful (e.g., iconic, pantomime, emblems; Lanyon and Rose, 2009). Although there is evidence that PwA are more successful at word retrieval when producing iconic gestures compared to other gesture types or no gesture (Akhavan et al., 2018), Lanyon and Rose (2009) found that not all PwA benefited from gesture during lexical retrieval, but those who did had phonological impairments (Lanyon and Rose, 2009). Another alternative explanation for the increased gesture use by PwA is that it serves a pragmatic function to signal to the listener that they are still searching to maintain their conversational turn (Beattie and Coughlan, 1999). Indeed, PwA produced more interactive gestures (i.e., gestures that coordinate dialogue such as flipping a hand to “pass” the turn to your interlocutor; Bavelas et al., 1992) than comparison participants both during spontaneous conversation and narrative retellings (de Beer et al., 2019).

#### Constraining Gesture

While some aphasia interventions encourage functional communication, others take a strict impairment-based approach in which they discourage forms of communication that may be compensatory in order to rehabilitate the target deficit (i.e., speech). One notable example of this is Constraint-Induced Aphasia Therapy (CIAT; Pulvermüller et al., 2001). In CIAT, communication is constrained to the spoken modality in an attempt to maximize spoken language recovery. CIAT aims to promote cortical reorganization (Taub et al., 2014) and is based on studies of limb rehabilitation in monkeys which found that constraining use of an unaffected limb forces use of a deafferented limb and improves mobility (Taub, 1976, 1980). Without intervention, the subjects developed “learned non-use” of the affected arm. This treatment was successfully extended to humans with impaired motor damage and limb use after neurological damage (Taub et al., 1993; Wolf et al., 2006, 2008) and termed Constraint-Induced Movement Therapy (CIMT). CIMT consists of four key components including (1) an intensive training schedule, (2) training behaviors through shaping, (3) a transfer package designed to generalize results beyond the research setting, and (4) discouraging compensatory behaviors (Taub et al., 2014). In theoretical extensions of this approach to aphasia, “learned non-use” results from compensatory or avoidance behaviors that include non-speech communication such as gestures and non-verbal sounds or the PwA remaining silent or allowing a caregiver to speak for them (Pulvermüller et al., 2001; Johnson et al., 2014).

Under the first constraint-induced aphasia protocol, people with chronic aphasia received 3 h of therapy every weekday for 2 weeks in a group-based language card game that resembles “Go Fish” and constrains communication to the verbal modality through the use of a barrier separating communication partners, the difficulty of stimuli used, explicit game rules provided by the therapist, and reinforcement of adherence to constraint rules (Pulvermüller et al., 2001). This protocol was subsequently modified to include a larger variety of expressive language exercises (e.g., repetition drills, picture description, role playing), increased intensity for verbal targets, and inclusion of a “transfer package” (Johnson et al., 2014), termed CIAT II. In a pilot of this most recent version of the intervention with four participants with moderate Broca’s aphasia, gesture was strongly discouraged, and therapists and caregivers were instructed not to respond to them. Overall, the participants reported improvement in their amount of verbal activity pre- to post-treatment and achieved large effect sizes for improvement in WAB-R aphasia quotients but without statistical significance for the small sample (Johnson et al., 2014).

Many versions of CIAT have been tested by different research groups with an overall positive impact on expressive communication (see Rose, 2013, for a summary of outcomes of constraint-induced language interventions); however, it is unclear whether the active ingredients of its success are related to gesture suppression or other factors such as the high intensity of treatment, group participation, caregiver training, and transfer package. Indeed, these studies are highly variable in the extent to which they constrain gesture and often not well described (Pierce et al., 2017). Some studies prohibited gesture use (Pulvermüller et al., 2001) and even strictly enforced spoken language by asking patients to sit on their hands if necessary (Maher et al., 2006; Kirmess and Maher, 2010; Martin et al., 2014). Others allowed gesture use as long as it was used to facilitate verbal language output (i.e., for self-cueing; Meinzer et al., 2007a, b; Difrancesco et al., 2012; Wilssens et al., 2015; Ciccone et al., 2016; Nickels and Osborne, 2016). In this view, PwA may use gesture to complement but not replace speech.

Thus, the use of gesture constraint has been interpreted and implemented very differently across CIAT studies, and it is important to consider its implications. While use of a barrier in the language game does not prevent the speaker from using gestures, it may implicitly decrease the amount of gestures they produce as people gesture less when their listener cannot see them (Alibali et al., 2001; Mol et al., 2011), and it prevents any gestures they do produce from being communicative to the listener. While it is a goal of CIAT for all communicative intentions to be completed verbally, this ignores the robust gesture literature on healthy adults that shows that gesture often naturally supplements speech, with people expressing unique information only in gesture, especially when talking about motor or spatial relations (Rauscher et al., 1996; Krauss, 1998; Feyereisen and Havard, 1999; Alibali et al., 2001; Hostetter and Alibali, 2007; Hostetter et al., 2007b). These gestures communicate to the listener but also may benefit the speaker in their organization, packaging, and conceptualization of information (Kita, 2000; Kita et al., 2017), beyond the self-cueing function described above. Furthermore, the act of consciously inhibiting gesture use may increase the cognitive load, especially for those with lower working memory capacity (Marstaller and Burianová, 2013; Gillespie et al., 2014) with implications for PwA for whom working memory deficits are common (Martin and Reilly, 2012).

Theoretical perspectives that propose that speech and gesture are tightly integrated processes predict that speech production might actually be hindered by gesture suppression. Indeed, in healthy adults, restricting gesture use has direct negative consequences on speech production; prohibiting gesture leads to impoverished speech content, resulting in less semantically rich descriptions of motor tasks (Hostetter et al., 2007a), decreased imagery (Rimé et al., 1984), fewer descriptions of perceptual-motor information (Alibali and Kita, 2010), and reduced speech fluency (Graham and Heywood, 1975; Rauscher et al., 1996; Morsella and Krauss, 2004). Conversely, explicitly encouraging gesture in healthy people improves recall (Goldin-Meadow et al., 2009), visuo-spatial problem solving (Chu and Kita, 2011) and perspective-taking in moral reasoning (Beaudoin-Ryan and Goldin-Meadow, 2014), highlighting the facilitative role of gesture production on various aspects of memory and reasoning. Importantly, two CIAT participants expressed frustration at being constrained to the verbal modality only (Maher et al., 2006), and the way gesture is treated in patient and caregiver training, a critical component of the intervention, may have long-term effects on how gesture is used with that communication partner. For example, training caregivers not to respond to communication attempts via gesture (e.g., Johnson et al., 2014) may result in increased communication breakdowns and frustration if gesture is taught as a mal-adaptive strategy. When gesture is allowed as a self-cueing mechanism for word retrieval, PwA may receive some benefit from spontaneous gesture; however, at best, this approach attenuates the potential of gesture for cognitive and communicative functions and at worst, may actually deny PwA access to the benefits of gesture in communication which may be critical ingredients of their language recovery. More research is needed to explore whether gesture can actually be leveraged to support language recovery in aphasia. The idea that gesture contributes to verbal “learned non-use” in aphasia is not empirically founded. Furthermore, constraining gesture has no theoretical support in current models of gesture production which propose that gesture and language represent an integrated (McNeill, 1992) or tightly coordinated system with both spoken language (de Ruiter, 2000; Kita, 2000) and cognition (Kita et al., 2017).

#### Encouraging Gesture

In contrast, other aphasia interventions encourage gesture use with the aim of either compensating for or restoring verbal communication (Rose, 2006). Recognition of aphasia as a disorder across modalities of communication (Hallowell and Chapey, 2001) has led to interventions incorporating the use of multiple modalities to facilitate recovery in which strengths in one modality may be leveraged to improve communication in another (Pierce et al., 2019). Indeed, many established aphasia intervention techniques take advantage of multiple modalities of communication including melodic intonation therapy (MIT; Sparks et al., 1974), Supported Conversation for Adults with Aphasia (SCA; Kagan et al., 2001), Promoting Aphasic Communicative Effectiveness (PACE; Davis, 2005), and Multiple-Modality Aphasia Treatment (M-MAT; Rose et al., 2013a). In addition to speech, these interventions may use drawing, music, symbol boards, and importantly, gesture. However, other treatments containing word-based cuing beyond speech (e.g., orthography) are also common and are not considered a multi-modality treatment by this definition (Pierce et al., 2019). Mutli-modality treatment approaches are thought to cue word retrieval and stimulate language and often take one of two aims: (a) improving speech or (b) improving total communication in which successful communication through any modality is encouraged (for a review, see Pierce et al., 2019). This latter approach trains functional communication tools that reduce communication breakdowns when word retrieval fails.

M-MAT and CIAT take different theoretical approaches to the potential interference or facilitation of gesture and other non-verbal modalities (see Rose, 2013 for a comparison of features of constraint vs. multi-modality interventions). However, functionally these two interventions share many common features that may help drive response to treatment (Pierce et al., 2017). Both interventions use group-based language games, are highly intensive, and rely on shaping to approximate desired communicative behaviors. However in M-MAT, there are no visual barriers, participants are given paper and pencil, and therapists provide cues and shaping for both verbal and multi-modal responses (Rose et al., 2019). M-MAT involves a cueing hierarchy where when naming pictures, participants make a verbal attempt first and if incorrect, the participant is next cued to produce an iconic gesture and re-attempt naming. Subsequent steps of the hierarchy involve clinician modeling of gesture, drawing, orthographic cues, and verbal repetitions (Rose et al., 2013a).

Direct comparisons of these interventions in a two-participant single-case design pilot study found a marginal advantage of M-MAT for the primary outcome measure of confrontation naming (Attard et al., 2013), but comparable effect sizes were found for both treatments in a group study of 11 PwA (Rose et al., 2013a). A systematic review of multi-modal and constraint-induced intervention approaches found limited empirical support for the superiority of either, although a meta-analysis of single-case experimental design studies favored multi-modal treatments (Pierce et al., 2017). The authors called for a more rigorous, direct comparison of these two approaches with explicitly described protocol for use of constraints or the types of multi-modality cueing used. This work is currently being undertaken by Rose and colleagues in a randomized controlled trial of constraint-induced or multi-modal personalized aphasia rehabilitation (COMPARE Trial; 2019). Currently, there is not enough evidence to support the use of gesture constraint, and its use should be cautioned against until more empirical evidence can evaluate any potential negative effects suppressing gesture may have on spoken language recovery in aphasia.

Likewise, more work is needed to explore the facilitatory effect that gesture may have in aphasia and the extent to which it corresponds to the functions of gesture observed in healthy adults. One obvious application is to study the effect of gesture on lexical retrieval in PwA. Murteira et al. (2019) report a lexical or semantic priming effect of observing congruent gestures on improved action picture naming in people without language impairments relative to observing an unrelated gesture or neutral stimulus prior to naming trials. In extending this study to PwA, this group found significant group differences for a facilitatory effect of observing congruent gestures for action verb naming for both naming accuracy and naming latencies (Murteira and Nickels, 2020). However, group results were more robust for naming latencies, and the effect of gesture varied considerably by individual. Studies looking at a role of gesture production for lexical retrieval of verb forms have had mixed results. A systematic review of gesture treatments for aphasia (Rose et al., 2013b) found that training gestures with verbal targets does improve word retrieval for trained stimuli. However, when comparing the effects of gesture + verbal treatment to verbal-only treatment on naming, some studies showed no advantage of gesture (Rodriguez et al., 2006; Rose and Sussmilch, 2008; Boo and Rose, 2011), but these studies had small samples of 2–4 participants. In contrast, Rose and Douglas (2001) did find a benefit for a subgroup of PwA: PwA had significantly improved picture naming when instructed to make a related iconic gesture but only if they had a primary phonological impairment as opposed to semantic or phonetic impairment. Similarly, in a study of 18 PwA, five produced more gestures during resolved word retrieval difficulties than unresolved, all of whom had phonological level impairments (Lanyon and Rose, 2009). These findings suggest that the facilitatory effect of gesture on lexical retrieval may depend on the individual PwA’s profile of relative strengths and deficits, and further work is needed to identify the participant and word-level factors that predict responsiveness to gesture in naming tasks in this heterogeneous patient population.

The large majority of gesture studies in aphasia have focused on using gesture to facilitate word retrieval but have left unexplored the many other communicative and cognitive functions of gesture. To our knowledge, at the time of this writing, no experimental studies in aphasia have examined how encouraging or constraining gesture affects the fluency of verbal output or whether listener perceptions of fluency are influenced by gesture use. Other open questions pertain to whether gesture facilitates planning or working memory capacity in the face of increased linguistic or processing demands, and whether gesture can be leveraged to improve learning and memory in aphasia which could lead to better retention for functional treatment stimuli. Importantly, in healthy people, producing gesture during learning not only improves recall of learned material but also leads to improved transfer of learning (Cook et al., 2013), the ultimate goal of successful language treatment. A single study examined the effect of producing gesture on word learning and memory in aphasia; 14 people with chronic mild aphasia learned novel labels for 30 manipulable objects by either gesturing and repeating target words or just repeating the words over 4 days (Kroenke et al., 2013). Recall was better for words that were encoded with gesture but only for people with phonological and working memory impairments. In fact, those with semantic impairments actually performed worse when producing gesture. These results accord with previous findings (Lanyon and Rose, 2009) that suggest that the benefits and function of gesture may depend on the individual’s aphasia profile, where those with phonological impairments rather than semantic impairments may have greater potential to benefit from gestural intervention. Indeed, intact semantic knowledge may be required to produce iconic gestures (Hadar and Butterwork, 1997; Cocks et al., 2013). These studies have important implications for the Lexical Retrieval Hypothesis (Krauss et al., 2000) which posits that gesture facilitates word retrieval through cross-modal priming at the phonological encoding stage. It may be more likely that iconic gesture operates on the cognitive processes involved in word retrieval by strengthening associations between preserved semantic representations. This is similar to the mechanism that underlies semantic approaches to language therapy such as semantic feature analysis (Efstratiadou et al., 2018) where words are retrieved via spreading activation of semantic associations, activating the lemma stage and, subsequently, the corresponding phonological representation (Maher and Raymer, 2004). Iconic gestures contain semantic features of their referents and reflect the distributed and experience-dependent conceptual representations in the brain (Kiefer and Pulvermüller, 2012). Thus, it may be this interaction between gesture and semantic memory that facilitates lexical retrieval. More work is needed to specify this mechanism to better predict treatment response and improve specificity of aphasia intervention. Future work should focus on exploring the cognitive and communicative functions of gesture in larger group studies of PwA to better identify individual and linguistic factors that may modulate the benefits of gesture.

### Cognitive-Communication Disorders

Cognitive-communication disorders are those for which domain general deficits in cognition such as attention, memory, problem solving, information processing, or executive function result in communication deficits. Given both the cognitive and communicative functions of gesture, it seems natural to study gesture in the context of cognitive-communication disorders and consider the ways in which gesture might uniquely reveal communication deficits or be leveraged to facilitate communication outcomes of people with brain injury and neurodegenerative diseases. However, cognitive-communication research has focused primarily on spoken language. It is an open question whether people with cognitive-communication disorders use gesture and benefit from gesture in the same way that healthy people do. Here we provide a brief overview of the deficits associated with RHD, TBI, and AD before reviewing the literature on gesture across these disorders.

#### Right Hemisphere Damage

Right hemisphere damage, often acquired after stroke, frequently results in a cognitive-communication disorder affecting pragmatics and discourse including a reduced ability to produce or comprehend emotional prosody, flat or monotone speech production, impaired comprehension of abstract or non-literal language, and impaired turn taking, topic maintenance, or eye contact (Blake, 2007, 2018; Blake et al., 2013). It is estimated that 50–68% of people with RHD exhibit at least one communication deficit (Blake et al., 2002; Côte et al., 2007). Critically, people with RHD also commonly experience visuospatial neglect (Kaplan and Hier, 1982; Bowen et al., 2013), which could impair their perception and production of gesture use.

#### Traumatic Brain Injury

Traumatic brain injury results from an external force that causes damage to the brain. In addition to anoxia, hemorrhages, edema, and seizures, the hallmark injury in TBI is diffuse axonal injury which decreases the integrity of white matter pathways, affecting the brain’s overall connectivity (Hayes et al., 2016). This diffuse neural injury results in heterogenous patterns of cognitive impairment across individuals with TBI. However, injury is common in the frontal and temporal lobes, producing deficits in executive functioning, processing speed, social cognition, and memory (Stuss, 2011). People with both mild (Leh et al., 2017) and moderate-severe TBI (Rigon et al., 2019, 2020) frequently demonstrate memory deficits. People with TBI also often have poor social outcomes (Wehman et al., 1993; Engberg and Teasdale, 2004; Kelly et al., 2008) which create barriers to community reintegration. Thus, assessment and treatment of cognitive-communication deficits is critical. Researchers have focused discourse analyses on documenting language impairments in coherence, cohesion, turn-taking, topic maintenance, and appropriateness (Bond and Godfrey, 1997; Coelho et al., 2002; Hough and Barrow, 2003; Davis and Coelho, 2004) and pragmatic skills (McDonald, 1993; McDonald and van Sommers, 1993; Turkstra et al., 1996; Bara et al., 2001). In addition to difficulties using and understanding language appropriately, people with TBI can also demonstrate impaired social cognition such as theory of mind and perspective taking deficits (Martín-Rodríguez and León-Carrión, 2010) and difficulty understanding irony (Martin and McDonald, 2005), sarcasm (Channon et al., 2005), and emotional affect (McDonald and Flanagan, 2004). Thus, people with TBI commonly have difficulty using non-verbal and extralinguistic cues to understand their communication partner’s needs and intentions. While some aspects of non-verbal communication including eye gaze (Turkstra, 2005) and facial affect recognition (Radice-Neumann et al., 2007; Rigon et al., 2017, 2018; Byom et al., 2019) have received independent attention, gesture has been relatively understudied.

#### Alzheimer’s Disease

Alzheimer’s disease is a neurogenerative disease characterized by gradually declining abilities in learning and memory and more observable impairments in connected speech and language as the disease progresses (Mueller et al., 2018). Although neuropathology is distributed throughout the brain in AD, the earliest and most severe pathology occurs in the medial temporal lobe, including the hippocampus (Hyman et al., 1990; Braak and Braak, 1991). This hippocampal atrophy has been linked to decreased memory performance in AD (Deweer et al., 1995; Laakso et al., 1995; Small et al., 1999; Kramer et al., 2004). Furthermore, hippocampal pathology in TBI has been linked to increased risk for later developing AD (Fleminger et al., 2003; Li et al., 2017). Given that memory deficits are hallmark to both TBI and AD, these populations provide a unique test of the reach of gesture in facilitating learning and memory. However, the diffuse nature of injury in these populations also make it difficult to isolate the effects of memory deficits alone.

Relative to aphasia, there has been significantly less research on gesture across these cognitive-communication disorders. The research that does exist has focused largely on characterizing gesture production in each population relative to healthy, non-injured comparison participants. In the next sections, we will review how researchers have examined gesture across cognitive-communication disorders by characterizing gesture production as well as work examining its functions in social communication and memory.

#### Characterizing Gesture Production

Given the prevalence of pragmatic deficits in RHD affecting paralinguistic (e.g., prosody) and non-verbal aspects of communication (e.g., eye contact), there has been some interest in whether RHD affects gesture production. Indeed, early case studies report a loss of emotional gesturing with two patients presenting with an “agestural state” (Ross and Mesulam, 1979). Further, there is evidence that people with RHD produce fewer iconic gestures overall than both healthy adults and people with aphasia (Hadar et al., 1998; Hogrefe et al., 2016) and that they produce fewer gestures in discourse samples with high emotional content compared to healthy adults (Cocks et al., 2007). Other studies have found an increase in self-touching movements such as grooming and scratching in RHD (Blonder et al., 1995; Cocks et al., 2007). One study found no difference in overall gesture production frequency between people with RHD and healthy adults in narrative discourse; however, significant positive correlations between the amount of gestures people with RHD produce and overall narrative competence suggest that gesture production may facilitate performance through domain general processes of attention and working memory (Akbıyık et al., 2018), lending support to the idea that gesture facilitates speaking and thinking by lightening the cognitive load (Goldin-Meadow et al., 2001).

Initial attempts to characterize gesture production in people with TBI have grouped gesture together with other aspects of non-verbal communication. These have mostly used rating scales to describe gesture as a subset of pragmatic communication. Aubert et al. (2004) used the Prutting and Kirchner: Pragmatic Aspects of Language (Prutting and Kirchner, 1987) qualitative scale to measure paralinguistic and non-verbal aspects of communication and found that facial expression, gaze functioning, and referential gesture were often impaired, especially in conversational discourse. Rousseaux et al. (2010) used a quantitative rating scale to measure aspects of non-verbal language and found that people with TBI in the rehabilitation stage (2–12 months post injury), but not the chronic stage (after 2 years), were globally impaired on non-verbal communication as well as understanding gestures (specifically relating to object shapes), but neither TBI group had deficits in producing gestures. Sainson et al. (2014) also used a rating scale to quantify gesture production in spontaneous conversation. They found that people with TBI were impaired in their frequency of gesture use relative to controls. Their gesture production was rated on a six-point scale from 0 (no impairment) to 5 (very severe impairment), however, this scale did not specify the direction of disruption (i.e., over- or under-production of gestures). In a case study examining gesture use and classifying gesture type in a single patient with multiple severe TBIs, the participant produced much fewer overall gestures and lower gesture rates than two healthy comparison participants, using only two iconic gestures in conversation (Sainson, 2007). These studies provide a cursory characterization of gesture production in TBI but lack a more quantitative approach and provide little insight into the types and communicative functions of gesture in this very heterogeneous population.

Considering gesture’s proposed role in lexical retrieval (Krauss et al., 2000), Kim et al. (2015) investigated the use of gesture by people with TBI who, though typically without aphasia, often demonstrate anomia (King et al., 2006; Hough, 2008). They analyzed the type and frequency of gestures that people with TBI produced during a confrontation naming task and found that people with TBI produced three times more co-speech gestures and hand movements than healthy comparison participants. Importantly, these hand movements included non-gesture movements that were unrelated to speech such as tapping, touching, and scratching. A significant negative correlation was also found where those with poorer performance on the word retrieval test produced more gestures and hand movements. This study provides an initial exploration of the association between gesture production and word retrieval in TBI but does not provide evidence for whether hand gesture has a facilitative function in resolving anomia.

There is a sparse literature exploring functions of gesture in pediatric TBI. Landry et al. (2004) found that infants (age 3–23 months) with history of severe TBI demonstrated reduced initiation of social interactions during play as well as reduced responsiveness to interactions initiated by the examiner compared to comparison participants; however, both groups were similar on indices of gestural and verbal communication. In contrast, Ewing-Cobbs et al. (2012) found that for children who had sustained a TBI before age 7, those children with moderate-severe TBI gestured more than those with mild TBI, possibly reflecting a developmental lag. Although much more work is needed to characterize gesture in pediatric TBI, there is additional support for a role of gesture for predicting and supporting language development from studies of children with pre- or perinatal unilateral brain lesions. The gesture use of children with these brain lesions at 18 months predicted later language development; those whose gesture use was in the typically developing range at 18 months developed vocabularies that did not differ from their typically developing peers whereas those whose gestures were below the typical developing range showed expressive and receptive vocabulary delay (Sauer et al., 2010). Thus, gesture could serve as in important diagnostic and therapeutic consideration for children at risk for communication disorders (Capone and McGregor, 2004). Furthermore, similar to healthy children (Rowe and Goldin-Meadow, 2009b), gesture-speech mismatches of children with these brain lesions later predicted simple (but not complex) sentence construction in speech (Özçalişkan et al., 2013). An additional study of children with perinatal unilateral brain lesions in kindergarten found that they benefited from seeing gesture, producing better structured narratives when retelling stories told by a narrator who used co-speech gestures compared to having seen and heard a story without gesture (Demir et al., 2014). These findings highlight an important role of gesture for scaffolding language and memory and evoking linguistic change for children with brain injuries.

Studies characterizing spontaneous gesture production in AD are also limited. An initial study found that the gestures of people with AD are more ambiguous and less complex with fewer semantic features, paralleling the use of empty speech in this population (Glosser et al., 1998). Similarly, Carlomagno et al. (2005) found that although people with AD produced a similar gesture rate to healthy comparison participants, they produced fewer iconic gestures. In contrast, Schiaratura et al. (2015) found that while people with AD had decreased quantity and quality of speech, their gestures were relatively unaffected.

In sum, the bulk of the work on gesture in cognitive-communication disorders has focused on characterizing gesture production in term of quantity and form and across different types of discourse elicitation tasks. While these disorders all have in common deficits in communication and cognition, there is variability as to the specific profiles across disorders and even within individuals who share the same diagnostic label. To better understand the shared and unique patterns of gesture production in individuals with neurogenic communication disorders we need considerably more gesture research and with larger samples sizes to account for known variability across and within disorders. Furthermore, use of experimental designs and protocols to examine gesture’s role in thinking, speaking, and remembering from the broader gesture literature in psychology and psycholinguistics would facilitate connection across literatures which is needed to advance understanding of the neural correlates of gesture in communication and cognition.

#### Gesture and Social Communication

Disruptions in social communication are common to all neurogenic communication disorders, but these impairments have perhaps been most studied and well-documented in TBI. Successful communication involves the integration and interpretation of both verbal and non-verbal signals. Healthy people automatically integrate information from a speaker’s speech and gesture (McNeill et al., 1994; Cassell et al., 1999) and use information from gesture to improve pragmatic understanding (Kelly et al., 1999). However, while gesture is an integral and often essential part of the communicative message, only a couple studies have examined the perception of gesture by people with TBI. Bara et al. (2001) examined whether people with TBI could use gestures to interpret communication acts of varying levels of complexity. Participants watched short silent movies in which the actors communicated only through gesture. Participants then chose a photograph representing the appropriate conclusion from a field of four. People with TBI were as successful as controls at using gesture to interpret simple and complex standard communication acts. However, people with TBI were significantly worse at interpreting gestures communicating deceit or irony. Evans and Hux (2011) examined whether people with TBI could integrate information from gesture with speech to accurately interpret indirect requests. Participants made predictions and interpretations after watching videos in which indirect requests were given verbally, with gesture-only, or with both speech and gesture together. Both people with TBI and healthy comparison participants interpreted indirect requests with greater accuracy when provided verbal and gesture information together than in either condition alone. However, people with TBI performed significantly worse than comparison participants on all conditions. These results indicate that people with TBI may be able to leverage gesture to reduce the deficit in their social communication performance and improve everyday communication, but more work is needed to understand this relationship. Furthermore, while people with TBI can successfully integrate verbal and non-verbal cues in a laboratory setting (Mutlu et al., 2019), little is known about how people with TBI rapidly integrate multiple cues, such as speech and gesture, for social interaction and decision-making in rich complex environments.

Studies examining listener perception of the gestures of people with TBI suggest that producing gesture may also facilitate social communication in this population. When judges rated a speaker with TBI on measures of pragmatic and communicative competence, “gesture appropriateness” was positively correlated with message effectiveness and ease of understanding, and hand/arm movements positively correlated with overall competence (Cannizzaro et al., 2011). Similarly, Jones and Turkstra (2011) found that when speakers with TBI narrated their accident stories, listeners perceived them as more charismatic when they gestured and indicated an increased likelihood of wanting to engage with them in a future conversation. These studies highlight gesture as a potential contributor to social communication outcomes in TBI. Given that neurogenic communication disorders more broadly disrupt social communication, research examining gesture’s role in social communication outcomes in RHD and AD is also warranted.

#### Gesture and Memory

Examining a link between gesture and memory in cognitive-communication disorders is important for several reasons: gesture plays a special role in promoting memory and learning; deficits in memory and learning are common across cognitive-communication disorders, and the success of all behavioral therapeutic interventions depends critically on memory and learning. Memory is not a unitary function but rather provides an account of our experiences and knowledge and includes the processes that support our encoding, consolidation, and retrieval of information with both temporary and long-term storage capacities. Memory is often divided into functionally and biologically distinct systems (Cohen and Squire, 1980; Eichenbaum and Cohen, 2001; Henke, 2010). Two primary systems that support long-term memory are the declarative and procedural memory systems. The declarative memory system, mediated by the hippocampus, supports acquisition of facts and world knowledge (semantic memory), and episodic events (episodic memory). In contrast, procedural memory, an aspect of non-declarative memory mediated by the basal ganglia, supports the acquisition of rules, habits, and skills. Both declarative and non-declarative memory interact closely with language acquisition (Ullman et al., 1997) and use (Brown-Schmidt and Duff, 2016; Duff and Brown-Schmidt, 2017). While not traditionally considered a cognitive-communication disorder, the study of a rare population of people with acquired bilateral hippocampal damage and amnesia who have severe declarative memory impairment but intact non-declarative memory has provided unique insights into understanding how memory systems support language use. The hippocampal declarative memory system supports relational binding and representational flexibility (Cohen and Eichenbaum, 1993) and has been found to underlie a variety of aspects of language use and processing (Duff and Brown-Schmidt, 2012, 2017). Only recently, there is a growing body of work characterizing the role of hippocampal declarative memory in gesture production. Hippocampal pathology is common to both AD (Hyman et al., 1984) and TBI (Bigler et al., 1996). Unlike AD, hippocampal pathology and memory deficits in amnesia are not progressive, but the results of these studies have important implications for cognitive-communication disorders resulting from memory deficits.

To test the role of the declarative memory system in gesture production, Hilverman et al. (2016) looked at spontaneous gesture production in a narrative task by people with focal, non-progressive, bilateral hippocampal damage and severe amnesia. They found that even though people with amnesia and healthy adults produced a similar amount of words in procedural and autobiographical narratives, people with amnesia had reduced gesture rates, producing significantly fewer gestures per word than their demographically healthy comparison participants. Critically, hippocampal amnesia does not produce motoric deficits that would interfere with the physical ability to produce gesture. However, while impoverished memory representations may not always affect the amount of speech produced, it does often impact its content: People with amnesia produce fewer episodic details relating to perceptual, temporal, and spatial information (Kurczek et al., 2015), and the words they produce are less imageable and concrete when producing narratives than healthy adults (Hilverman et al., 2017). These findings suggest that the declarative memory system contributes to rich and imageable information conveyed in both speech and gesture and that when impaired, in any population, may result in impoverished speech and gesture.

While these findings suggest that declarative memory impairments lead to impoverished gesture production, other studies demonstrate that the spontaneous gestures people with amnesia produce are communicative and uniquely reveal information about their knowledge and experiences. For example, when healthy people share knowledge (i.e., common ground) with a communication partner, they attenuate both their speech and gesture use in parallel, producing both fewer words and gestures (Campisi and Özyürek, 2013; Galati and Brennan, 2013; Hoetjes et al., 2015; Hilliard and Cook, 2016; but see Jacobs and Garnham, 2007; Holler and Wilkin, 2009 for two exceptions). There is evidence that multiple memory systems support the acquisition and use of common ground (Brown-Schmidt and Duff, 2016). When designing communication for a child compared to an adult listener, people with amnesia adapt more in gesture than speech relative to healthy adults; healthy adults increased both the number of words and gestures they produced when demonstrating how to do everyday tasks to a child (e.g., how to change a lightbulb) whereas people with amnesia increased their gesture use for the child above and beyond the changes they made in the number of words produced (Clough et al., in review)^2^. People with amnesia also reflect shared knowledge in gesture but somewhat inconsistently in speech; in a collaborative referential barrier game in which the participant accrues incremental common ground with a partner through multiple rounds of a matching game, people with amnesia arrived at concise shared spoken labels with the partners (Duff et al., 2006); however, they did not consistently use definite references like the healthy adults (Duff et al., 2011). In gesture, people with amnesia signaled common ground by producing fewer visible gestures above the barrier over the course of the game as shared knowledge and familiarity increased (Hilverman et al., 2019). This parallels research in healthy adults that shows that when speakers share common ground with a listener, their produce gestures that are lower in the visual field (Hilliard and Cook, 2016). Thus, despite greatly reduced explicit recall for episodic information, the gestures of people with declarative memory impairments can reflect their knowledge and experiences.

These studies provide important evidence that speech and gesture can dissociate and, under certain conditions, may be differentially supported by distinct memory systems. Although people with amnesia produce fewer gestures during narrative discourse, indicating that gestures stem from declarative memory representations, their gestures uniquely communicate information about their knowledge states and experiences, even for information they may be unable to verbalize and declare. This supports the idea that gesture reflects implicit knowledge (Broaders et al., 2007), and therefore, may engage the non-declarative memory system in some contexts. Indeed, the gestures of people with Parkinson’s disease, which affects the basal ganglia system supporting non-declarative, or procedural memory, do not reflect their prior experiences performing a motor task (Klooster et al., 2015). Gestures themselves may also be considered implicit in that both speakers and listeners rarely consciously attend to them, and yet, both healthy adults and people with amnesia integrate information from co-speech gesture into their narrative retellings of a story, suggesting that gesture-speech integration does not depend on the hippocampal declarative memory system (Hilverman et al., 2018a). Furthermore, gesture often reflects implicit transitional knowledge states by communicating information that is not yet verbally accessible (Goldin-Meadow et al., 1993). If non-declarative memory supports gesture, then it is possible that gesture can be leveraged to improve recall in people with declarative memory impairments. To test this idea, participants with amnesia completed a novel word-learning task (a task they are profoundly impaired on) by learning word-object associations while producing gesture, observing gesture, or without gesture; although the participants were unable to freely recall the labels when tested, they demonstrated above chance recognition memory for labels but only when they produced gesture during learning (Hilverman et al., 2018b). This research in individuals with hippocampal amnesia offers exciting new insights into the relationship between memory and gesture, however, as of yet, it has largely unexplored implications for people with cognitive-communication disorders.

## Discussion

Gesture provides a unique window into a speaker’s mind and provides a direct link between cognition and communication. However, despite a robust literature on the functions of gesture for thinking, speaking, and remembering in healthy adults, gesture has been relatively underexplored in populations with neurogenic communication disorders. Here we assert that gesture is not just an accessory to the language system, but rather an integral partner in communication. A broader approach to the study of language provides insights into these rich communicative contexts. Here, language is a dynamic process that is locally constructed between communication partners and leverages multiple modalities of information, including gesture. Minimally, we have reviewed literature showing that gesture has essential communicative functions above and beyond speech, and therefore, researchers studying neurogenic communication disorders should work to also characterize the consequences of these disorders on the gestural modality. Indeed, as a field, we know much less about gesture than spoken language in these disorders as well as knowing less about gesture than even other non-verbal aspects of communication such as eye-gaze or facial affect recognition. Much of the research that exists on gesture in these populations has focused on characterizing spontaneous gesture production but often from an atheoretical perspective. Studying gesture in populations with impairments in language and cognition provides a unique opportunity to test hypotheses generated by various theoretical accounts in the gesture literature in healthy adults which suggests that gesture provides cognitive and linguistic benefits. Indeed, despite well-documented deficits in memory and social communication after cognitive-communication disorders, researchers have not explored whether patterns of brain injury or cognitive deficit predict gesture use or if gestures can improve memory and communicative function in these individuals.

Of all the functions of gesture described here, perhaps the most exciting is the potential benefit of gesture on learning and memory and the implications that this might have for clinical practice. The success of all behavioral therapy depends on the ability of the patient to learn and remember the targeted skills. Yet, rather than incorporating gesture into our interventions, some therapy protocols have inhibited it. This seems counterproductive in light of the potent role of gesture in learning and memory. Rather than discouraging gesture production, it may be more useful to consider the synergistic nature of speech and gesture and explore ways to leverage gesture to achieve various intervention goals across disciplines. To date, the bulk of the theoretical and empirical work on co-speech gesture has been from a cognitive or psychological perspective rather than a neural correlates perspective. Thus, while we know a lot about the cognitive and communicative benefits of gesture, we know less about the neural mechanisms that support them. Applying the psychological literature of gesture to neurogenic communication disorders not only has the potential to improve treatment, but also provides an opportunity to generate and advance theories of co-speech gesture that are psychologically and biologically plausible.

While this review identifies several gaps in the neurogenic communication disorder literature, it highlights an exciting opportunity to consider neurogenic communication disorders from a new perspective. Our hands shape and actively alter our own learning and display traces of that learning in conversation, reflecting our prior experiences and depicting knowledge even for things the speaker may not be explicitly aware of or cannot yet communicate in speech. In addition to supporting learning and memory, gesture facilitates the exploration of ideas especially when it comes to visuo-spatial problem solving and complex reasoning. Yet, we know little about how gesture interacts with cognition in clinical populations, and this is critical to fully understand language, cognition, and communication, and its disorders. Thus, gesture deserves more of our attention in the study of neurogenic communication disorders. Future research should systematically assess the impact of cognitive and communication disorders on gesture production in larger group studies as well as empirically testing the functions of gesture for language use and social cognition. Such research would shed light on the untapped potential of gesture in understanding and rehabilitating neurogenic communication disorders.

## Author Contributions

SC and MD planned the scope and content of the review. SC wrote the initial version of the manuscript. MD contributed to the final version of the manuscript in writing and editing. Both authors contributed to the article and approved the submitted version.

## Conflict of Interest

The authors declare that the research was conducted in the absence of any commercial or financial relationships that could be construed as a potential conflict of interest.

## Footnotes

1 We use the term “speech” from the larger gesture literature, referring to the content of spoken language. It is not in reference to articulation, coordination, or planning associated with the motor act of producing speech.

2 The role of the hippocampus in perspective-taking: implications for cognitive-communication disorders. Paper Presented at the International Cognitive-Communications Disorders Conference, Orange, CA.
